
JOURNAL INFORMATION
==============================
NLM Title Abbreviation: ScientificWorldJournal
Journal ID: ScientificWorldJournal
NLM Journal ID: 101131163
Journal ID: TSWJ

The Scientific World Journal

ISSN: 2356-6140
EISSN: 1537-744X
Publisher: Wiley

ARTICLE INFORMATION
==============================
PMCID: PMC6084106
PMID: 30147620
DOI: 10.1100/tsw.2001.240
Article ID: 913918
Article version: 1
Subjects: Short Report

Predominant Identification of RNA-Binding Proteins in Fas-Induced Apoptosis by Proteome Analysis

Rudel Thomas *
Dimmler Christiane
Siejak Frank
Thiede Bernd
Max-Planck-Institute for Infection Biology, Dept. of Molecular Biology, Schumannstr. 21/22, 10117 Berlin, Germany
*Thomas Rudel: rudel@mpiib-berlin.mpg.de
Publication date: 2001
Electronic publication date: 2001 Dec 5
Volume: 1
First page: 143
Last page: 143
Copyright: Copyright © 2001 Frank Siejak et al.
Copyright year: 2001
License: This is an open access article distributed under the Creative Commons Attribution License, which permits unrestricted use, distribution, and reproduction in any medium, provided the original work is properly cited.
License URL: https://creativecommons.org/licenses/by/3.0/

Funding: Bundesministerium für Bildung und Forschung

==============================
Miami *Nature Biotechnology* Short Reports

*TheScientificWorld* (2001) 1 (S3), 143SR

ISSN 1532-2246; DOI 10.1100/tsw.2001.240

### PREDOMINANT IDENTIFICATION OF RNA-BINDING PROTEINS IN FAS-INDUCED APOPTOSIS BY PROTEOME ANALYSIS

Thomas Rudel*, Christiane Dimmler, Frank Siejak, and Bernd Thiede

Max-Planck-Institute for Infection Biology, Dept. of Molecular Biology, Schumannstr. 21/22, 10117 Berlin, Germany
* rudel@mpiib-berlin.mpg.de

**INTRODUCTION.** Apoptotic signaling is accompanied by post-translational modification of proteins. The most prominent modification is the cleavage of proteins by caspases, the main effectors of apoptotic signaling (1). On the basis of our previously published proteome of Jurkat T cells (2), we now used the proteome technology to identify proteins that were modified during apoptosis.

**METHODS.** Jurkat T cells were induced to undergo apoptosis by treatment with Anti-Fas-IgM. The proteins were separated by a large gel 2-DE technique (gel size 30 cm x 23 cm), stained as described (2) and identified by MALDI-MS or ESI-MS. cDNAs of identified proteins were isolated and modifications were verified in vitro using recombinant proteins.

**RESULTS.** We identified 84 proteins by proteome analysis that were modified during apoptosis. These proteins exhibited either a different molecular mass, or/and a different isoelectric point (pI) or were present in reduced amounts in apoptotic cells. Strikingly, several of the identified proteins contained the RNP motive known to be responsible for RNA binding. We investigated the mechanism underlying the apoptotic modification of one of these proteins, the 54 kD nuclear RNA-binding protein ($p54^{nrb}$) in detail. Using *in vitro* cleavage assay at least three caspase cleavage sites were identified. Surprisingly, of the three caspase cleavage sites identified in $p54^{nrb}$ only one was not sensitive to RNA binding. Thus, binding of RNA to proteins is a possible parameter for their sensitivity to caspase cleavage.

**DISCUSSION.** Here we demonstrated the usefulness of proteome approaches to identify proteins modified during apoptosis. Proteins thus far not shown to be modified during apoptosis were identified and the modification of some of these proteins was verified *in vitro* to depend on caspase cleavage. An unexpected result of our screen was the identification of a relatively large number of proteins containing a RNA-binding motive. Our data showed for the first time that the splicing factor $p54^{nrb}$ belongs to this class of RNA-binding proteins cleaved by caspases. It is presently unknown how caspase cleavage modifies the function of $p54^{nrb}$. However, the observation that the presence of RNA affected at least two of the three caspase cleavage sites identified in $p54^{nrb}$ suggested a role of the modification by caspases *in vivo*.

**ACKNOWLEDGEMENT.** This work was supported by the Bundesministerium für Bildung und Forschung (BMBF).

### REFERENCES.

1. Thornberry, N.A. and Lazebnik, Y. (1998) Science 281**,** 1312-1316
2. Thiede, B., Siejak, F., Dimmler, C., Jungblut, P.R., and Rudel, T. (2000) Electrophoresis 21**,** 2713-2720
